# Genetic relationship between Hashimoto`s thyroiditis and papillary thyroid carcinoma with coexisting Hashimoto`s thyroiditis

**DOI:** 10.1371/journal.pone.0234566

**Published:** 2020-06-30

**Authors:** Ohoud Subhi, Hans-Juergen Schulten, Nadia Bagatian, Roa'a Al-Dayini, Sajjad Karim, Sherin Bakhashab, Reem Alotibi, Alaa Al-Ahmadi, Manar Ata, Aisha Elaimi, Saad Al-Muhayawi, Majid Mansouri, Khalid Al-Ghamdi, Osman Abdel Hamour, Awatif Jamal, Jaudah Al-Maghrabi, Mohammed Hussain Al-Qahtani

**Affiliations:** 1 Center of Excellence in Genomic Medicine Research, Department of Medical Laboratory Technology, Faculty of Applied Medical Sciences, King Abdulaziz University, Jeddah, Saudi Arabia; 2 Department of Biochemistry, Faculty of Sciences, King Abdulaziz University, Jeddah, Saudi Arabia; 3 Center of Innovation in Personalized Medicine, Department of Medical Laboratory Technology, Faculty of Applied Medical Sciences, King Abdulaziz University, Jeddah, Saudi Arabia; 4 Department of Otolaryngology, Head and Neck Surgery, Faculty of Medicine, King Abdulaziz University, Jeddah, Saudi Arabia; 5 Department of Surgery, King Faisal Specialist Hospital and Research Center, Jeddah, Saudi Arabia; 6 Department of Pathology, Faculty of Medicine, King Abdulaziz University, Jeddah, Saudi Arabia; 7 Department of Pathology, King Faisal Specialist Hospital and Research Center, Jeddah, Saudi Arabia; Universidade do Porto Faculdade de Medicina, PORTUGAL

## Abstract

Hashimoto's thyroiditis (HT) is present in the background of around 30% of papillary thyroid carcinomas (PTCs). The genetic predisposition effect of this autoimmune condition is not thoroughly understood. We analyzed the microarray expression profiles of 13 HT, eight PTCs with (w/) coexisting HT, six PTCs without (w/o) coexisting HT, six micro PTCs (mPTCs), and three normal thyroid (TN) samples. Based on a false discovery rate (FDR)-adjusted *p*-value ≤ 0.05 and a fold change (FC) > 2, four comparison groups were defined, which were HT *vs*. TN; PTC w/ HT *vs*. TN; PTC w/o HT *vs*. TN; and mPTC *vs*. TN. A Venn diagram displayed 15 different intersecting and non-intersecting differentially expressed gene (DEG) sets, of which a set of 71 DEGs, shared between the two comparison groups HT *vs*. TN **∩** PTC w/ HT *vs*. TN, harbored the relatively largest number of genes related to immune and inflammatory functions; oxidative stress and reactive oxygen species (ROS); DNA damage and DNA repair; cell cycle; and apoptosis. The majority of the 71 DEGs were upregulated and the most upregulated DEGs included a number of immunoglobulin kappa variable genes, and other immune-related genes, e.g., CD86 molecule (*CD86*), interleukin 2 receptor gamma (*IL2RG*), and interferon, alpha-inducible protein 6 (*IFI6*). Upregulated genes preferentially associated with other gene ontologies (GO) were, e.g., *STAT1*, *MMP9*, *TOP2A*, and *BRCA2*. Biofunctional analysis revealed pathways related to immunogenic functions. Further data analysis focused on the set of non-intersecting 358 DEGs derived from the comparison group of HT *vs*. TN, and on the set of 950 DEGs from the intersection of all four comparison groups. In conclusion, this study indicates that, besides immune/inflammation-related genes, also genes associated with oxidative stress, ROS, DNA damage, DNA repair, cell cycle, and apoptosis are comparably more deregulated in a data set shared between HT and PTC w/ HT. These findings are compatible with the conception of a genetic sequence where chronic inflammatory response is accompanied by deregulation of genes and biofunctions associated with oncogenic transformation. The generated data set may serve as a source for identifying candidate genes and biomarkers that are practical for clinical application.

## Introduction

Hashimoto’s thyroiditis (HT), also known as chronic lymphocytic thyroiditis, is induced by an autoimmune response where immune cells produce autoantibodies that target thyroid antigens, in first instance thyroid peroxidase (TPO) and thyroglobulin (TG) leading to atrophy and transformation of thyroid cells [1, 2]. This autoimmune reaction is caused by infiltrating autoreactive T lymphocytes and other components of the immune defense system that constitute an inflammatory infiltrate, which under chronic conditions leads to thyroiditis and hypothyroidism [2, 3]. Risk factors for HT include environmental factors, such as exposure to ionizing radiation, carcinogenic chemicals, iodine deficiency, and infections, as well as non-environmental risk factors, such as genetic susceptibility, that can be linked in certain cases with a history of inherited autoimmune diseases [4, 5]. Genome-wide association studies (GWAS) have identified a number of genetic susceptibility loci that are associated with autoimmunity, especially with development of thyroid autoantibodies [6, 7]. A systematic review on studies, carried out in their majority on Caucasian populations, reported that incidence of hypothyroidism, which is commonly associated with HT, is 350/100,000/year in women and 80/100,000/year in men [8]. Although controversial discussed, several studies including a meta-analysis on fine-needle aspiration (FNA) and thyroidectomy studies reported HT as a risk factor for papillary thyroid carcinoma (PTC) development, and this observation was mainly based on the proportionally higher incidence of PTC with (w/) HT compared to PTC without (w/o) HT [9, 10].

Thyroid carcinoma (TC) is the most common malignant endocrine cancer with a worldwide increasing incidence and especially PTC as the most common type of thyroid carcinoma contributes to this prevalence [11]. Micro PTCs (mPTCs) are ≤ 10 mm in size and regarded as a low risk and indolent subtype of PTC [12–14]. The mortality rates in TC are approximately between 0.20 and 0.60/100,000/year in women and 0.20 and 0.40/100,000/year in men [11]. The female prevalence observed in most of the thyroid lesions is also dominant in PTC w/ HT where cancer incidence is approximately five times higher in females than in males [15]. A meta-analysis on 71 observational studies reported that PTCs w/ HT are more likely to be multifocal but in general have favorable clinicopathological features including less extrathyroid extension, lymph node and distant metastases, and recurrence than PTCs w/o HT [16].

Reactive oxygen species (ROS), which are DNA damaging agents, are a physiological component of thyroid cell function and have elevated expression in HT. For example, a study on public-accessible RNA sequencing (RNA-seq) and microarray data provided genomic evidence that up-regulated genes related to ROS metabolism and apoptosis were significantly more prevalent in PTC w/ HT compared to PTC w/o HT [17]. This led to the notion that higher ROS levels in HT may contribute to PTC development. An immunohistochemistry (IHC) study on PTC-associated tumor markers suggested that in HT, under prolonged exposure to chronic inflammation, a fraction of follicular epithelia show PTC-like nuclear aberrations that might lead to oncogenic transformation [18]. Collaborating with this, a number of studies identified molecular events common to both HT and TC. For instance, loss of heterozygosity (LOH) in the DNA damage repair gene 8-oxoguanine DNA glycosylase (*OGG1*) has been frequently found in HT and PTCs, but rarely in benign goiter [19]. An IHC study detected, in comparison to normal thyroid tissue, elevated expression of pAKT and the two isoforms AKT1, AKT2 in HT and in well-differentiated TC w/ or w/o HT [20]. In contrast, tumor suppressor PTEN showed positive expression in normal thyroid tissue and HT but only heterogeneous/absent expression in TC w/ HT. Another IHC study detected positive TP63 expression in HT and PTC, which led to the suggestion that both entities share a pathobiological relationship [21]. Furthermore, ret proto-oncogene (*RET*)/PTC rearrangements have been detected in a FISH and real-time PCR study at low frequencies in follicular cells of HT, which led to the assumption that these fusions may contribute to early stages of PTC development [22]. In contrast, *BRAF* mutations that are almost mutually exclusive to *RET*/PTC rearrangements, are not present in HT w/o coexisting PTC and are comparably more prevalent in PTC w/o HT [23].

The primary aim of the present study was to identify genes and biofunctions, which are shared between HT and PTC w/ HT. Findings should help to better understand the molecular etiopathogenesis between HT and PTC w/ HT and support further assessment of candidate genes for clinical application.

## Material and methods

### Tumor samples

For microarray expression analysis, 33 thyroid tissue specimens derived from partial or entire thyroidectomies were preserved in RNAlater (Qiagen Inc., Hilden, Germany) and selected for this study according to the histopathology reports. Age of patients ranged between 14 and 67 years with a mean age of 38.7 years, SD = 14.1 (S1 Table). Five of the six micro PTCs (mPTCs) coexisted with HT. The study was approved by the Biomedical Ethics Unit, Faculty of Medicine, King Abdulaziz University, (approval number: 358–10), and the Institutional Review Board of King Faisal Specialist Hospital and Research Center (approval number: IRB2010-07). Written informed consent from the donors or the next of kin was obtained for the use of samples in research. Microarray CEL files from three normal thyroid (TN) samples were downloaded from a website of the microarray vendor (Thermo Fisher Scientific, Waltham, MA) and served as control.

### Sample processing

Total RNA was isolated from the samples using the RNeasy Mini-Kit according to the manufacturer’s protocol (Qiagen Inc.). RNA concentration was determined using the NanoDrop 2000 spectrophotometer (Thermo Fisher Scientific). RNA integrity was assessed in three cases by gel electrophoretic RNA separation and in 30 cases by using the 2100 Bioanalyzer (Agilent Technologies, Santa Clara, CA). The RNA integrity number (RIN) in these 30 samples ranged between 5.9 and 9.3 (mean, 7.66) and included only RNA in the acceptable quality range between nearly intact and slightly degraded RNA (chosen RIN cutoff value for clinical tumor samples, 5.0). The processed RNA samples were hybridized to HuGene 1.0 ST microarrays according to established protocols [24]. The labeled and washed microarrays were scanned on a GeneChip Scanner 3000 7G and CEL files were generated by the GeneChip Command Console Software AGCC (Thermo Fisher Scientific).

### BRAF mutational status

Sequence analysis of BRAF exon 15 on PCR products, by using either a genomic DNA template or a cDNA template, was essentially reported earlier [24, 25].

### Raw data analysis

The CEL files were imported into Partek Genomic Suite version 6.6 (Partek Inc., Chesterfield, MO) where expression data were normalized using the robust multi-array average (RMA) method in the default settings. A principal component analysis (PCA) was then conducted on the categorized samples and subsequently, lists of significantly differentially expressed genes (DEGs) (false discovery rate (FDR)-adjusted p-value < 0.05 and fold change (FC) > 2) were computed for the four comparison groups HT *vs*. TN; PTC w/ HT *vs*. TN; PTC w/o HT *vs*. TN; and mPTC *vs*. TN. Statistical assessment of a comparison group HT w/ PTC *vs*. HT w/o PTC generated no DEGs (FDR-adjusted p-value < 0.05 and FC > 2). From DEGs, represented by multiple probe sets, the set with the highest FC was selected for further processing. To generate intersecting and non-intersecting DEG sets from different comparison groups, a web-based Venn diagram tool was employed (http://bioinformatics.psb.ugent.be/webtools/Venn/) (S2 Table). Where applicable, the Ensemble genome browser and HUGO Gene Nomenclature Committee (HGNC) multi symbol checker were utilized to annotate gene IDs according to the approved symbols [26, 27]. An expression dendrogram was generated using the online web tool Heatmapper and employing average linkage clustering and Spearman`s rank correlation for distance measurement on the gene set [28]. The generated microarray data set has been deposited at the NCBI’s Gene Expression Omnibus (GEO) under accession number GSE138198 and includes renormalized sample files from submission GSE54958.

### Gene ontology (GO)

GO annotations of the generated data set were compiled from different resources (S3 Table). Genes related to immune and inflammatory functions were retrieved from the online GO resource AmiGo2 v2.5.12 (search strings: immun, interleukin, histocompat, inflamm, cytokine, chemokine, interferon, lymphocyte) [29]. Cell cycle genes were retrieved from the The Molecular Signatures Database (msigdb) v7.0 [30] and a vendor resource (https://www.qiagen.com/nl/resources/resourcedetail?id=0ee18e97-d445-4fd7-9aa4-0ef4bece124f&lang=en). Apoptosis genes were derived from AmiGo2 (search string: apoptosis) and a vendor resource (https://www.qiagen.com/ch/resources/resourcedetail?id=e5252c51-7513-44a0-b66d-927c53e0eeb2&lang=en). DNA damage and DNA repair genes were compiled from a publication resource [31], a database of DNA damage response-related proteins (DDRprot) [32], and a vendor resource (https://www.qiagen.com/sg/resources/resourcedetail?id=909a9567-91e0-43d9-96c7-a7700182c3af&lang=en). ROS-related genes were retrieved from AmiGo2 (search strings: reactive oxygen species, oxidative stress) and a vendor resource (https://www.qiagen.com/us/products/discovery-and-translational-research/pcr-qpcr/qpcr-assays-and-instruments/mrna-incrna-qpcr-assays-panels/rt2-profiler-pcr-arrays/?catno=PAHS-065Z#geneglobe). A comprehensive list of genes mutated in differentiated thyroid cancer was acquired from the cBioPortal for Cancer Genomics [33]. Gene IDs of ncRNAs were downloaded from a HGNC webpage [27]. All files from the vendor and HGNC were accessed in August 2019.

### Pathway and network analysis

Biological significance of DEGs was further interpreted using the Ingenuity Pathway Analysis software and its pathway Knowledge Base (IPA; build version 485516M; Qiagen Inc.). Analysis settings included direct and indirect molecular relationships. Fisher`s exact test p-values indicated significant relationships between analyzed data set molecules and frameworks prebuilt or generated *de novo* by the software application. The Molecule Activity Predictor was employed to predict expression effects of a molecule on other pathway/network molecules as noted in the prediction legends of the respective figures. The overlap percentage in canonical pathways indicates the relative number between uploaded and predefined pathway molecules. Network analysis was performed to explore significance of fit, expressed as a score, between uploaded data set molecules and networks related to specific diseases and functions. The upstream analysis module was employed to assess, by utilizing z-scores, in how far differences in target gene expression are affected by upstream regulators.

## Results

**Fig 1:**
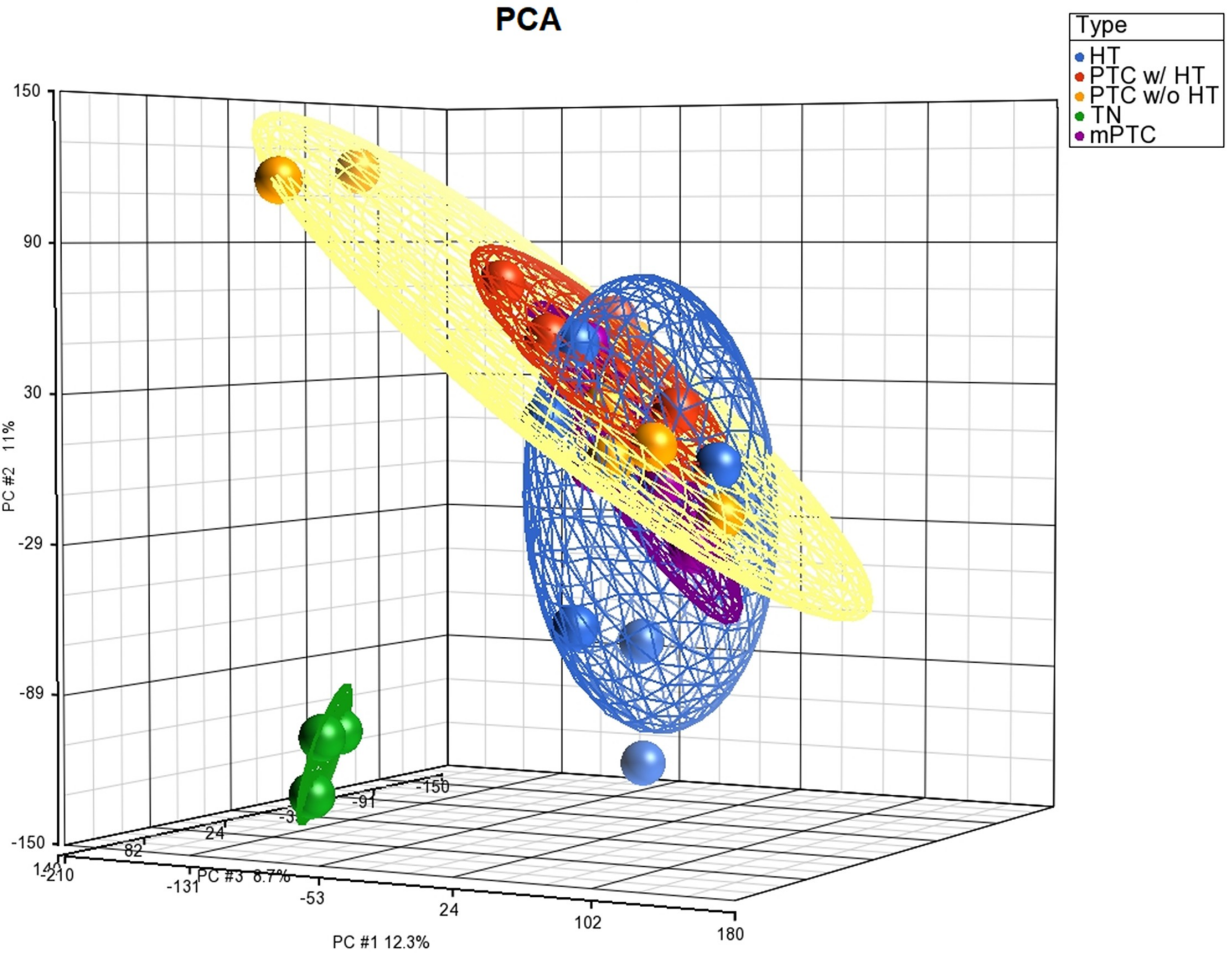
PCA 3D scatter plot that represents a dimensional transformation of expression data where the distance-related measure indicates the expression similarity between samples. Each sample is marked by a dot and each group of samples by a 95% confidence ellipsoid. Color classification is noted in the color scheme.

**Fig 2:**
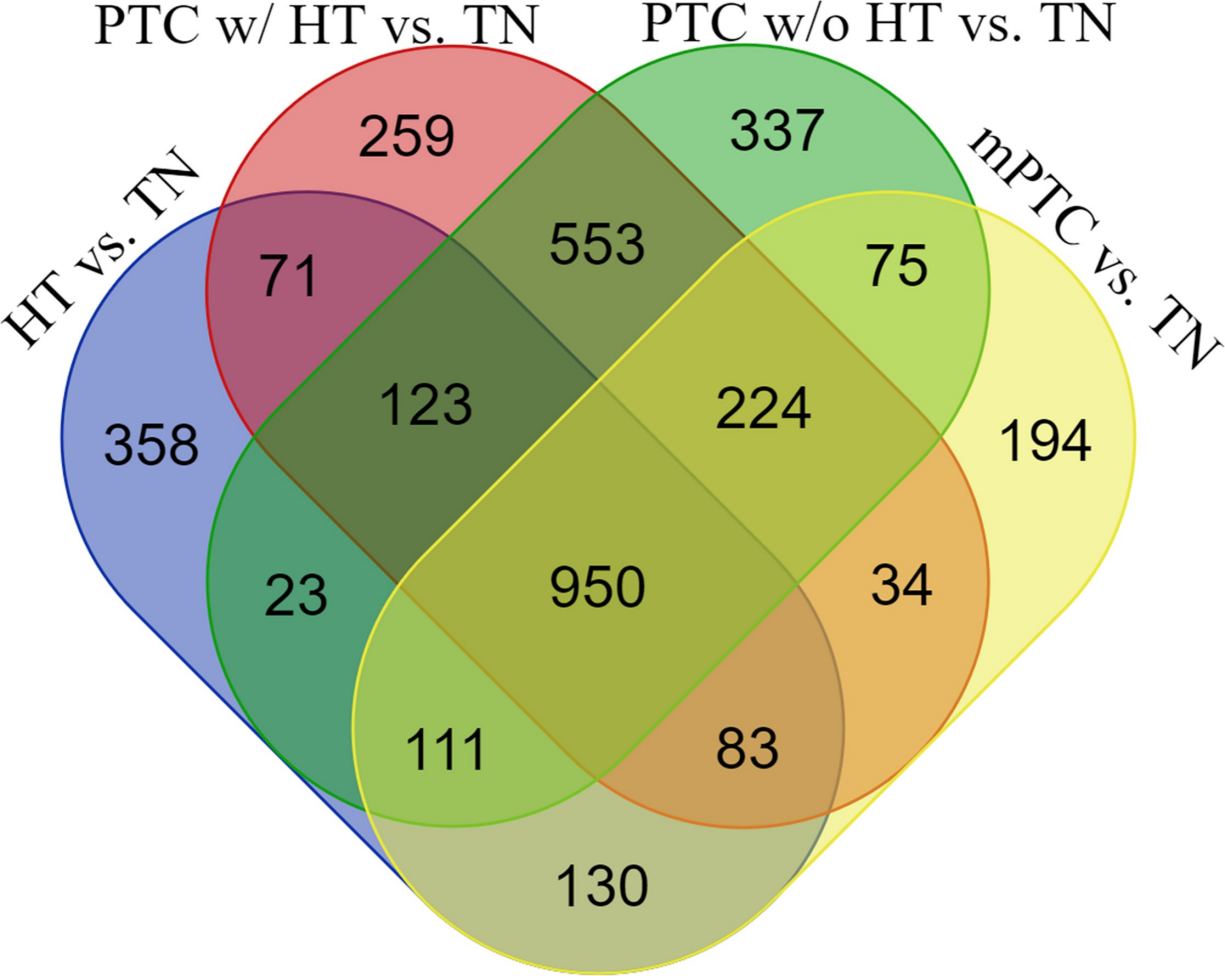
Venn diagram illustrates intersecting and non-intersecting DEG sets derived from the four comparison groups HT *vs*. TN; PTC w/ HT *vs*. TN; PTC w/o HT *vs*. TN; and mPTC *vs*. TN. Number of DEGs is indicated in each section.

To narrow down the genetic relationship between HT and PTC w/ HT, in reference to PTC w/o HT, mPTC, and TN, we analyzed the molecular expression profiles of a case series consisting of 13 HTs, eight PTCs w/ HT, six PTCs w/o HT, six mPTCs, and three TN samples. Demographic and clinicopathological data of the case series are listed in S1 Table. All tumors, except one, were stage I cases and a V600E BRAF mutation was revealed in eight cases. Similarity of expression profiles of the different groups of cases is presented in a PCA 3D scatter plot demonstrating that expression profiles of the different thyroid disease groups partially overlap whereas TN samples clearly cluster separately (Fig 1). Four comparison groups of DEGs were generated, i.e., HT *vs*. TN; PTC w/ HT *vs*. TN; PTC w/o HT *vs*. TN; and mPTC *vs*. TN. A Venn diagram displays the number of annotated DEGs, which are either unique for a comparison group or are shared between two or more comparison groups (Fig 2) (S2 Table). In our study, we focused in first instance on the 71 DEGs, which were shared between the two comparison groups HT *vs*. TN ⋂ PTC w/ HT *vs*. TN. In further instance, we focused on the non-shared 358 DEGs derived from the comparison group HT *vs*. TN and on the 950 DEGs, which are shared between the four comparison groups, i.e., HT *vs*. TN ⋂ PTC w/ HT *vs*. TN ⋂ PTC w/o HT *vs*. TN ⋂ mPTC *vs*. TN. Top canonical pathways and networks that are most significantly related to the 71, 358, and 950 DEG sets are to varying degrees enriched in immunogenic functions (Table 1).

**Table 1:** Top pathways and networks of the 71, 358, and 950 DEG sets.

Category			*p*-Value	Overlap [%]	Score
**Top canonical pathways**					
	**HT *vs*. TN ∩ PTC w/ HT *vs*. TN (71 DEG set)**				
		Production of nitric oxide and reactive oxygen species in macrophages	2.22E-05	3.0	
		T helper cell differentiation	3.29E-05	6.2	
		IL-7 signaling pathway	1.00E-04	4.7	
		Interferon signaling	1.46E-04	8.3	
		Neuroinflammation signaling pathway	1.85E-04	2.0	
	**HT *vs*. TN (358 DEG set)**				
		Th1 pathway	7.04E-08	10.5	
		Th1 and Th2 activation pathway	6.02E-07	8.0	
		Systemic lupus erythematosus signaling	3.17E-06	7.0	
		iCOS-iCOSL signaling in T helper cells	1.26E-05	8.7	
		T cell receptor signaling	1.47E-05	8.5	
	**HT *vs*. TN ∩ PTC w/ HT *vs*. TN ∩ PTC w/o HT *vs*. TN ∩ mPTC *vs*. TN (950 DEG set)**				
		Calcium signaling	5.56E-07	13.2	
		Cdc42 signaling	7.96E-07	16.2	
		Mitochondrial dysfunction	1.65E-06	13.9	
		Antigen presentation pathway	3.64E-06	30.0	
		Actin cytoskeleton signaling	6.11E-05	10.6	
**Top networks related to diseases and functions**					
	**HT *vs*. TN ∩ PTC w/ HT *vs*. TN (71 DEG set)**				
		Cellular function and maintenance, hematological system development and function, immunological disease			37
		Cell cycle, cellular development, connective tissue development and function			37
		DNA replication, recombination, and repair, cell death and survival, cellular compromise			19
		Gastrointestinal disease, immunological disease, organismal injury and abnormalities			8
		Cancer, cell cycle, cell death and survival			2
	**HT *vs*. TN (358 DEG set)**				
		DNA replication, recombination, and repair, nucleic acid metabolism, small molecule biochemistry			43
		Cell cycle, cell morphology, cellular assembly and organization			38
		Cellular development, cellular growth and proliferation, hematological system development and function			34
		Lymphoid tissue structure and development, tissue morphology, cellular development			31
		Cellular growth and proliferation, cell death and survival, cancer			29
	**HT *vs*. TN ∩ PTC w/ HT *vs*. TN ∩ PTC w/o HT *vs*. TN ∩ mPTC *vs*. TN (950 DEG set)**				
		Hereditary disorder, organismal injury and abnormalities, skeletal and muscular disorders			49
		Amino acid metabolism, small molecule biochemistry, hematological disease			39
		Cell-to-cell signaling and interaction, hereditary disorder, neurological disease			39
		RNA post-transcriptional modification, auditory disease, neurological disease			39
		RNA post-transcriptional modification, cell cycle, DNA replication, recombination, and repair			37

**Fig 3:**
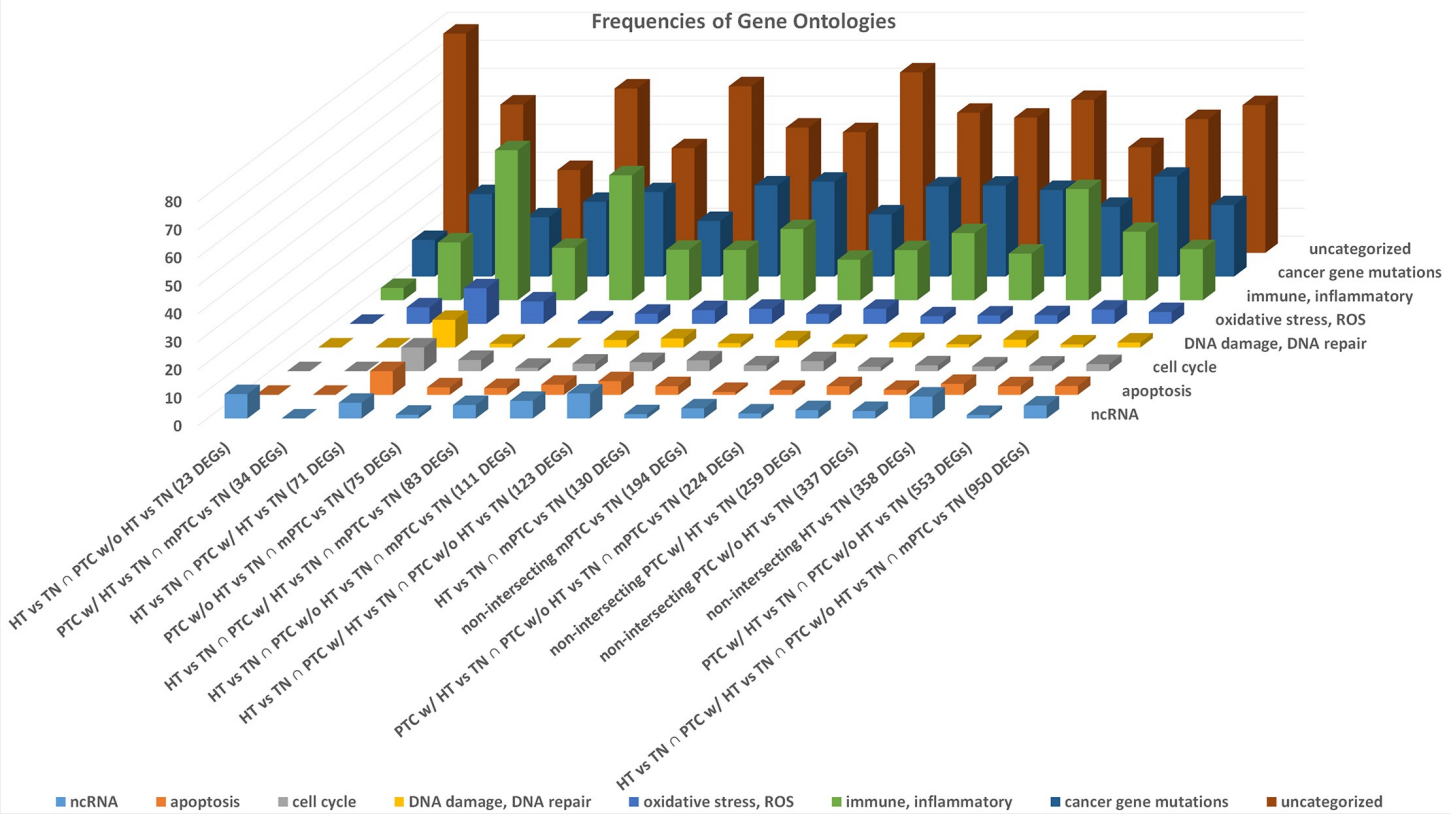
Bar chart displaying for each of the 15 intersecting and non-intersecting DEG sets, derived from the four comparison groups, the relative number of DEGs, which are categorized into different GOs. The 71 DEGs from the intersection of HT *vs*. TN ∩ PTC w/ HT *vs*. TN reveal the relatively highest number of DEGs in the categories immune and inflammatory functions; oxidative stress and ROS; DNA damage and DNA repair; cell cycle; and apoptosis. Concomitantly, the 71 DEGs harbor the relatively lowest number of uncategorized GOs. A number of genes were classified into more than one GO category. ncRNA, non-coding RNA.

For all 15 DEG sets, a GO classification in various categories was performed to generate an approximate overview of the functional implications of the sets. The 71 DEGs from the intersection of HT *vs*. TN ∩ PTC w/ HT *vs*. TN harbored the relatively highest number of DEGs related to immune and inflammatory functions; oxidative stress and ROS; DNA damage and DNA repair; cell cycle; and apoptosis (Fig 3).

### Analysis of the 71 DEG set from the intersection of HT *vs*. TN ∩ PTC w/ HT *vs*. TN

**Fig 4:**
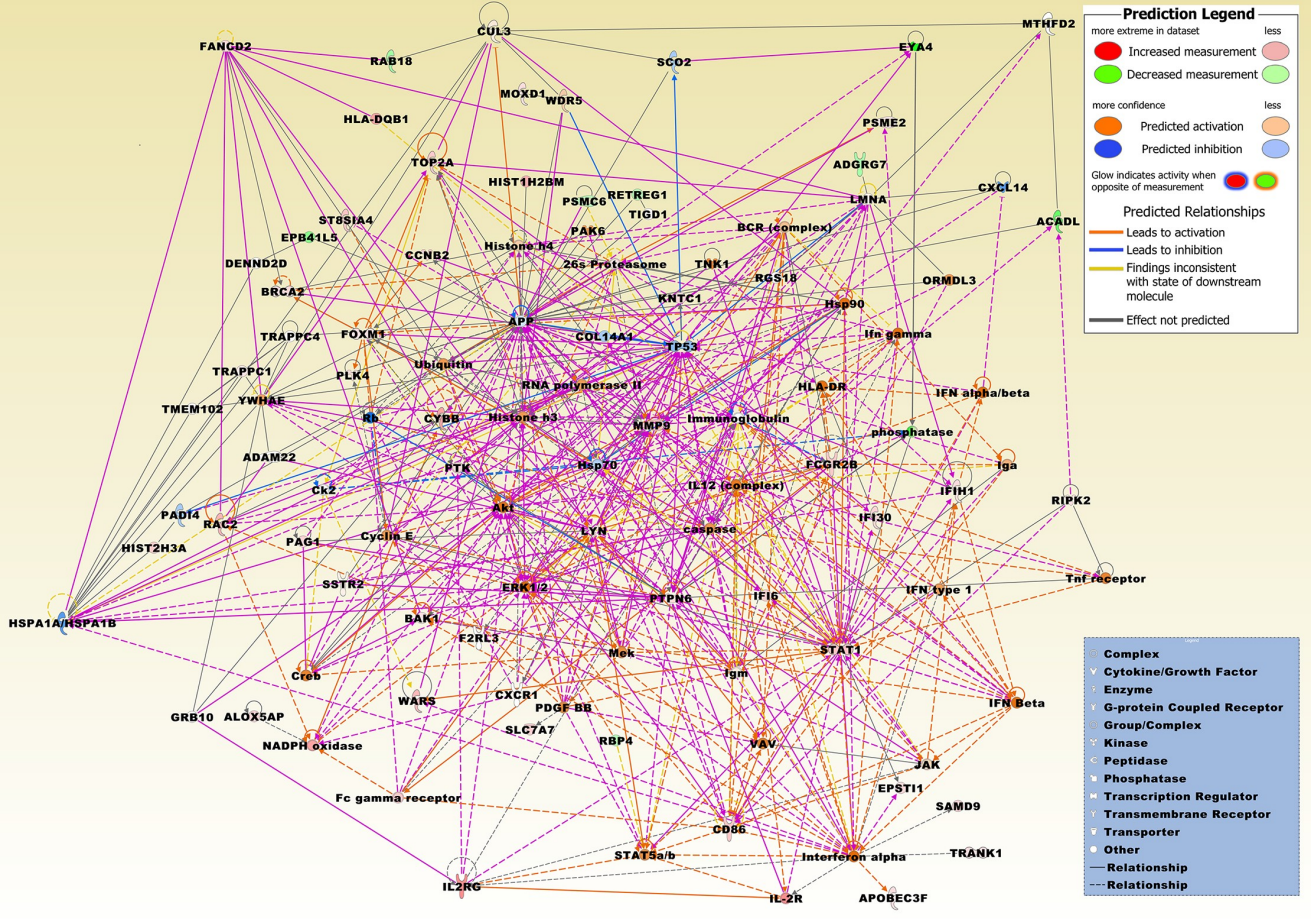
The top three merged networks that are based on the 71 DEGs from the intersection of HT *vs*. TN ∩ PTC w/ HT *vs*. TN. Upregulated molecules from the DEG set include ALOX5AP, APOBEC3F, BAK1, BRCA2, CCNB2, CD86, CYBB, EPSTI1, FANCD2, FCGR2B, FOXM1, HIST1H2BM, HIST2H3A, HLA-DQB1, IFI30, IFI6, IFIH1, IL2RG, KNTC1, LYN, MMP9, MOXD1, PAG1, PLK4, PSME2, PTPN6, RAC2, RIPK2, SAMD9, SLC7A7, ST8SIA4, STAT1, TOP2A, TRANK1, TRAPPC1, and WARS. Downregulated molecules from the DEG set include ACADL, ADGRG7, EPB41L5, EYA4, PSMC6, RAB18, RBP4, and RETREG1. Integrative molecules from the IPA Knowledge Base comprise 26s Proteasome, ADAM22, Akt, APP, BCR (complex), caspase, Ck2, COL14A1, Creb, CUL3, CXCL14, CXCR1, Cyclin E, DENND2D, ERK1/2, F2RL3, Fc gamma receptor, GRB10, Histone h3, Histone h4, HLA-DR, Hsp70, Hsp90, HSPA1A/HSPA1B, IFN alpha/beta, IFN Beta, Ifn gamma, IFN type 1, Iga, Igm, IL12 (complex), IL-2R, Immunoglobulin, Interferon alpha, JAK, LMNA, Mek, MTHFD2, NADPH oxidase, ORMDL3, PADI4, PAK6, PDGF BB, phosphatase, PTK, Rb, RGS18, RNA polymerase II, SCO2, SSTR2, STAT5a/b, TIGD1, TMEM102, Tnf receptor, TNK1, TP53, TRAPPC4, Ubiquitin, VAV, WDR5, and YWHAE.

**Fig 5:**
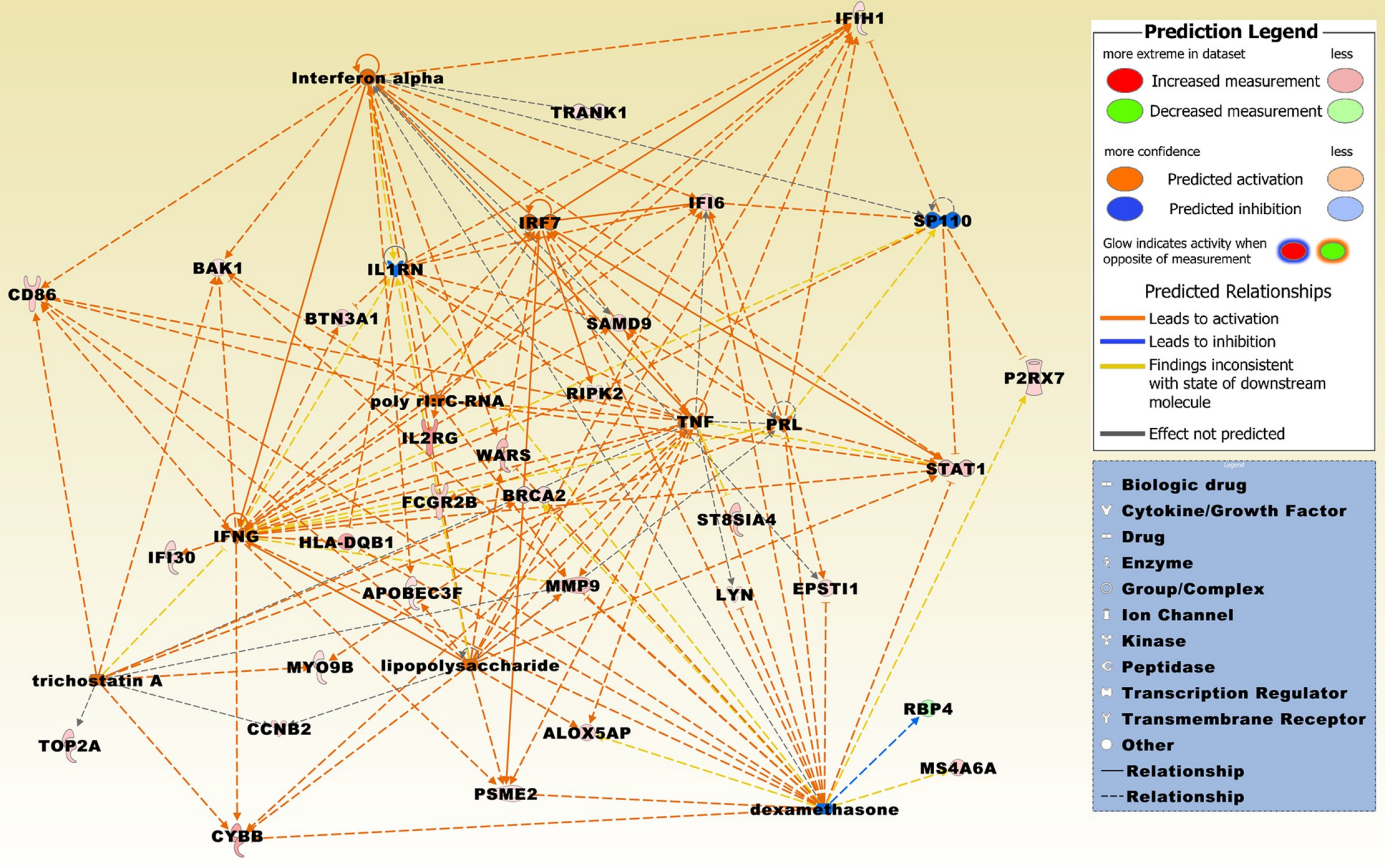
Upstream analysis on the set of 71 DEGs. The activated upstream regulators (z-score ≥ 2) comprise IFNG, interferon alpha, TNF, lipopolysaccharide, IRF7, PRL, poly rI:rC-RNA, trichostatin A and the inhibited upstream regulators (z-score ≤ -2) comprise IL1RN, dexamethasone, and SP110. Molecules derived from the 71 DEG set comprise ALOX5AP, APOBEC3F, BAK1, BRCA2, BTN3A1, CCNB2, CD86, CYBB, EPSTI1, FCGR2B, HLA-DQB1, IFI30, IFI6, IFIH1, IL2RG, LYN, MMP9, MS4A6A, MYO9B, P2RX7, PSME2, RBP4, RIPK2, SAMD9, ST8SIA4, STAT1, TOP2A, TRANK1, and WARS.

Sixty of the 71 DEGs were comparably upregulated whereas only 11 DEGs were downregulated. The most significantly upregulated genes included a number of immunoglobulin kappa variable genes, i.e., *IGKV3D-11*, *IGKV1-13*, *IGKV1D-16*, *IGKV1D-33*, *IGKV1D-42*, and *IGKV1D-43*. Further overexpressed immune-related genes comprised MHC class II gene *HLA-DQB1*, immune checkpoint gene CD 86 molecule (*CD86*), interleukin 2 receptor, gamma (*IL2RG*), and interferon, alpha-inducible protein 6 (*IFI6*). Overexpressed genes, preferentially related to other GOs, included, e.g., ras-related C3 botulinum toxin substrate 2 (Rac family small GTPase 2 (*RAC2*), ST8 alpha-N-acetyl-neuraminide alpha-2,8-sialyltransferase 4 (*ST8SIA4*), tryptophanyl-tRNA synthetase 1 (*WARS1*, alias WARS), STAT1, membrane spanning 4-domains A6A (*MS4A6A*), BCL2 antagonist/killer 1 (*BAK1*), polo-like kinase 4 (*PLK4*), cytochrome b-245 beta chain (*CYBB*), and phosphoprotein membrane anchor with glycosphingolipid microdomains 1 (*PAG1*), *MMP9*, *TOP2A*, *BRCA2*, forkhead box M1 (*FOXM1*), and H2B clustered histone 14 (*H2BC14*). Among the most comparably underexpressed genes were EYA transcriptional coactivator and phosphatase 4 (*EYA4*), adhesion G protein-coupled receptor G7 (*ADGRG7*), acyl-CoA dehydrogenase, long chain (*ACADL*), and erythrocyte membrane protein band 4.1 like 5 (*EPB41L5*). A merged network and an upstream analysis, which are most significantly related to the 71 DEGs, are displayed in Figs 4 and 5, respectively, and a hierarchical cluster analysis of the 71 DEG set is illustrated in S1 Fig.

### Analysis of the non-intersecting 358 DEG set derived from the comparison group HT *vs*. TN

**Fig 6:**
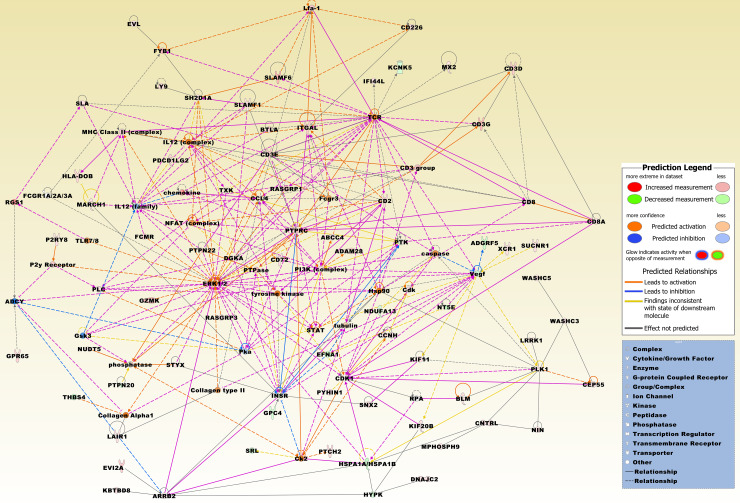
The top three merged networks that are based on the non-intersecting 358 DEGs derived from the comparison group HT *vs*. TN. Upregulated molecules from the DEG set include ABCC4, ADAM28, ARRB2, BLM, BTLA, CCL4, CCNH, CD2, CD226, CD3D, CD3E, CD3G, CD72, CD8A, CDK1, CEP55, CNTRL, DGKA, DNAJC2, EVI2A, EVL, FCMR, FYB1, GPR65, GZMK, HLA-DOB, IFI44L, ITGAL, KBTBD8, KIF11, KIF20B, LAIR1, LRRK1, LY9, MARCH1, MPHOSPH9, MX2, NIN, NUDT5, P2RY8, PDCD1LG2, PLK1, PTCH2, PTPN22, PTPRC, PYHIN1, RASGRP1, RASGRP3, RGS1, SH2D1A, SLA, SLAMF1, SLAMF6, SNX2, STYX, SUCNR1, TXK, WASHC3, WASHC5, and XCR1. Downregulated molecules include ADGRF5, EFNA1, GPC4, HSPA1A/HSPA1B, HYPK, INSR, KCNK5, NDUFA13, NT5E, PTPN20, SRL, and THBS4. Integrative molecules from the IPA Knowledge Base comprise ADCY, caspase, CD3 group, CD8, Cdk, chemokine, Ck2, Collagen Alpha1, Collagen type II, ERK1/2, FCGR1A/2A/3A, Fcgr3, Gsk3, Hsp90, IL12 (complex), IL12 (family), Lfa-1, MHC Class II (complex), NFAT (complex), P2y Receptor, phosphatase, PI3K (complex), Pka, PLC, PTK, PTPase, RPA, STAT, TCR, TLR7/8, tubulin, tyrosine kinase, and Vegf.

In the 358 DEG set, approximately 80% of the genes were upregulated. In contrast to the 71 DEG set, the most upregulated genes included also several immunoglobulin heavy variable genes, i.e., *IGHV3-35*, *IGHV3-43*, *IGHV3-52*, *IGHV3-73*, *IGHV3-74*, *IGHV4-59*, *IGHV5-78*, *IGHV3OR15-7*, *IGHV3OR16-7*, *IGHV3OR16-8*, and *IGHV1OR15-1*. Other overexpressed immune-related genes comprised a number of chemokines, i.e., *CXCL13*, *CCL19*, *CCL4L2*, *CCL4*, and furthermore, the CD3 chain genes *CD3G*, *CD3E*, and *CD3D*, the non-receptor type 22 (lymphoid) (*PTPN22*), and the immune checkpoint genes *CD28* and programmed cell death 1 ligand 2 (*PDCD1LG2*). Overexpressed genes related to other GOs included, e.g., BCL2-related protein A1 (*BCL2A1*), marker of proliferation Ki-67 (*MKI67*), and the tumor suppressor Ras association domain family member 6 (*RASSF6*). Among underexpressed genes were thrombospondin 4 (*THBS4*), ephrin-A1 (*EFNA1*), insulin-like growth factor 1 receptor (*IGF1R*), adhesion G protein-coupled receptor F5 (*ADGRF5*), and the membrane-bound *MMP14*. A merged network, which is most significantly related to the 358 DEG set, is displayed in Fig 6.

### Analysis of the 950 DEG set from the intersection of all four comparison groups

**Fig 7:**
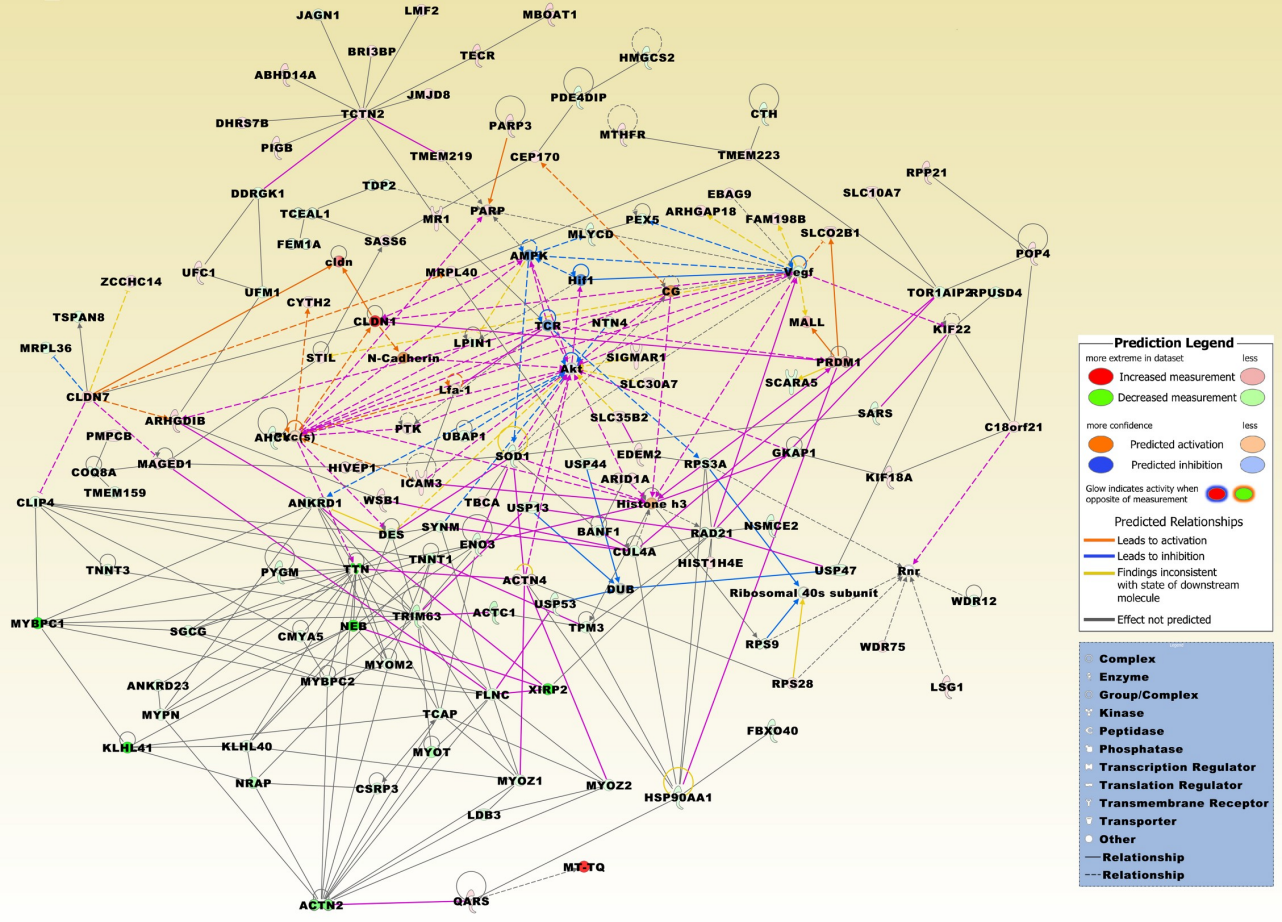
The top three merged networks that are based on the intersecting 950 DEGs of all four comparison groups, i.e., HT *vs*. TN ∩ PTC w/ HT *vs*. TN ∩ PTC w/o HT *vs*. TN ∩ mPTC *vs*. TN. Upregulated molecules include ABHD14A, ACTN4, ARHGAP18, ARHGDIB, ARID1A, BRI3BP, C18orf21, CEP170, CLDN1, CLDN7, CYTH2, DHRS7B, EBAG9, EDEM2, FAM198B, HIST1H4E, HIVEP1, ICAM3, JMJD8, KIF18A, KIF22, LMF2, LSG1, MAGED1, MALL, MBOAT1, MR1, MRPL40, MTHFR, MT-TQ, PARP3, PIGB, PMPCB, POP4, PRDM1, QARS, RPP21, RPS28, SASS6, SIGMAR1, SLC10A7, SLC30A7, SLC35B2, SLCO2B1, STIL, TBCA, TCTN2, TECR, TMEM219, TMEM223, UFC1, WDR75, WSB1, and ZCCHC14. Downregulated molecules from the DEG set include ACTC1, ACTN2, AHCY, ANKRD1, ANKRD23, BANF1, CLIP4, CMYA5, COQ8A, CSRP3, CTH, CUL4A, DDRGK1, DES, ENO3, FBXO40, FEM1A, FLNC, GKAP1, HMGCS2, HSP90AA1, JAGN1, KLHL40, KLHL41, LDB3, LPIN1, MLYCD, MRPL36, MYBPC1, MYBPC2, MYOM2, MYOT, MYOZ1, MYOZ2, MYPN, NEB, NRAP, NSMCE2, NTN4, PDE4DIP, PEX5, PYGM, RAD21, RPS3A, RPS9, RPUSD4, SARS, SCARA5, SGCG, SOD1, SYNM, TCAP, TCEAL1, TDP2, TMEM159, TNNT1, TNNT3, TOR1AIP2, TPM3, TRIM63, TSPAN8, TTN, UBAP1, UFM1, USP13, USP44, USP47, USP53, WDR12, and XIRP2. Integrative molecules from the IPA Knowledge Base comprise Akt, AMPK, CG, cldn, DUB, Hif1, Histone h3, Lfa-1, N-Cadherin, PARP, Pkc(s), PTK, Ribosomal 40s subunit, Rnr, TCR, and Vegf.

In the 950 DEG set, the number up- and downregulated genes was nearly the same. Among genes with highest overexpression were several small nucleolar RNAs, e.g., *SNORA71D*, *SNORD45A*, *SNORA16A*, *SNORD29*, *SNORD13P3*, *SNORD13*, and *SNORA22*. Other overexpressed genes comprised a number of chemokines, including the chemokine (C-X-C motif) ligands *CXCL10* and CXCL16. Among important oncogenes were poly (ADP-ribose) polymerase family, member 3 (*PARP3*), *TGFB1*, *TP53*, and EPH receptor B2 (*EPHB2*). Underexpressed genes included, e.g., superoxide dismutase 1, soluble (*SOD1*), superoxide dismutase 2, mitochondrial (*SOD2*), RAD21 homolog (S. pombe) (*RAD21*), *BRCA1*, and vascular endothelial growth factor D (*VEGFD*). A merged network, which is most significantly related to the 950 DEG set, is displayed in Fig 7.

## Discussion

This is one of the first microarray expression studies that established gene expression profiles simultaneously in HT, PTCs w/ HT, PTCs w/o HT, mPTCs, and TN samples. By applying a categorization scheme, we were able to generate a comprehensive overview of DEGs shared or non-shared between different types of thyroid lesions with a focus on HT and PTC w/ HT. In the intersection of PTC w/ HT *vs*. TN ∩ PTC w/o HT *vs*. TN we found for instance a strong downregulation of *TPO*, which is in line with its well-characterized low expression levels in PTC [34].

Although the link between ROS, inflammation and cancer has been extensively discussed, the underlying molecular genetics are not fully explored yet [35]. An expression analysis study on a publicly deposited RNA-seq data set focused on detection of ROS-related genes in PTCs w/ HT *vs*. PTCs w/o HT and identified 33 genes that were upregulated in PTCs w/ HT compared to nine in PTCs w/o HT [17]. Furthermore, the study found a considerable overlap of the identified upregulated ROS genes with a publicly deposited microarray expression data set. The immune-related expression pattern of genes in PTC was investigated in a number of studies. An protein expression study detected endogenous IgGγ and IgGκ expression in the majority of PTC and colocalization of IgGγ with complement proteins led to the suggestion that immune complexes are formed that may promote PTC cells from escaping the immune response against tumor antigens [36]. The hypothesis that the immunogenic link between HT and PTC may stimulate their synchronous appearance was also discussed in detail [37].

### Upregulated genes of the 71 DEG set from the intersection of HT *vs*. TN ∩ PTC w/ HT *vs*. TN

The top upregulated genes in the 71 DEG set included a number of immunoglobulin kappa variables. Expression of light chains has been demonstrated in the vast majority of HT in a flow cytometry study where most cases showed a κ/λ light chain ratio in the range of non-lymphomatous polyclonality [38]. In a preclinical model system, tumor growth could be reduced by inhibiting free light chain-mediated mast cell activation [39]. The MHC class II gene *HLA-DQB1* has been reported in a microarray expression assay to be differentially expressed between PTC and follicular variant of PTC (FVPTC) [40]. The immunoglobulin superfamily member CD86 is expressed on antigen-presenting cells (APCs) and involved in B-T cell interaction and activation. Flow cytometry analysis in blood of patients with early onset Graves’ disease and HT detected increased CD86 expression in thyroid-reactive B cells [41].

IL2RG is a critical signaling component of a number of interleukin receptors and has been described in a systems biology approach as a factor involved in T cell differentiation signaling during progression of PTC [42]. IFI6 is implicated in apoptosis regulation and exhibits a role in hepatitis C virus infection [43]. The polysialyltransferase *ST8SIA4* has been identified in a multiplex meta-analysis of RNA expression datasets as one of the top deregulated genes associated with various immune dysfunctions [44]. STAT1 is implicated in signaling of IFN and a number of interleukins and therefore represents a critical factor of the normal immune response. In addition, STAT1 participates to the morphology and function of the thyroid [45]. An IHC study in HT indicated that tyrosine-phosphorylated STAT1 staining was associated with a thyrocytes to oncocytes transformation as well as with a fraction of infiltrating lymphocytes and germinal macrophages [46]. Of notice, STAT1 constitutes a molecular target for anti-inflammatory treatment [47]. The putative tumor suppressor gene *PAG1* encodes a transmembrane adaptor protein that binds to the tyrosine kinase Csk and participates in the negative control of T cell receptor (TCR) signaling [48]. The cytoplasmic enzyme WARS1 catalyzes the ligation of tryptophan to its cognate tRNA during the translation process and beyond this, exerts diverse biological functions in innate immunity, angiogenesis, and IFNG signaling [49]. A microarray mRNA expression profiling study in several major blood cell subtypes derived from normal individuals identified a comparable overexpression of *MS4A6A* in monocytes/platelets [50]. The function of MS4A6A in autoimmune-related thyroid lesions remains to be elucidated.

The small GTPase Rac2 is involved in various aspects of host defense including integrin and immunoreceptor signaling, polarization to M2 macrophages and generation of ROS [51–53]. *Rac2* knockout mice demonstrated a pronounced impairment in tumor growth, angiogenesis, and metastasis [52]. The membrane-bound CYBB is part of the catalytic core of an NADPH oxidase multi-protein complex that produces a phagocytic respiratory burst by generating superoxide in response to pathogens and various other stimuli [53]. The proapoptotic BAK1 protein is a BCL2 family member and an inducer of the intrinsic apoptotic pathway. All three genes, encoding for RAC2, CYBB, and BAK1, were reported to be deregulated among ROS-related genes in an RNA-seq data set analysis between PTC w/ HT and PTC w/o HT [17]. Overexpression of the zinc-dependent metalloproteinase MMP9 has been previously associated with invasion of follicular ML-1 thyroid cancer cells [54]. Furthermore, *in vitro* assays demonstrated that MMP9 expression is necessary for the serine/threonine kinase ROCK1 to promote PTC cell invasion [55]. TOP2A is a critical factor for sister chromosome segregation and is expressed at highest levels in G2 and M cell cycle phases. The enzyme induces double-stranded DNA breaks, which activate autoimmune regulator (Aire)-associated proteins leading to increased and efficient gene transcription [56]. The serine/threonine protein kinase PLK4 is involved in initiating centriole assembly. Its reversible inhibition may represent a prospective anticancer therapy [57]. Upregulation of BRCA2 is likely a DNA repair response mechanism to elevated DNA damage under chronic ROS exposure. The transcription factor FOXM1 is involved in many crucial biological processes and specifically related to cell cycle progression and cancer evolution [58, 59]. FOXM1 overexpression was detected by IHC in 28.4% of PTCs [60]. Further *in vitro* and *in vivo* study found that the FOXM1 inhibitor thiostrepton inhibited PTC cell line xenograft tumor growth and this was accompanied by downregulation of FOXM1, MMP9, and MMP2. H2BC14, alias HIST1H2BM, has been identified as one of several molecules that participate in progression through the cell cycle and that are regulated by the p53-p21-DREAM-E2F/CHR pathway [59].

### Downregulated genes of the 71 DEG set from the intersection of HT *vs*. TN ∩ PTC w/ HT *vs*. TN

Downregulation of *EYA4* expression in cells of epithelial origin is consisting with its tumor suppressor function in these entities [61]. ADGRG7, alias GPR128, is a receptor of the adhesion GPCR family, whose functional relevance in thyroid lesions is virtually unknown; however, promoter studies indicated that ESRα and SP1 play a critical role in positively regulating expression of the receptor [62, 63]. ACADL is a peroxisomal enzyme and is part of a family of enzymes known to be regulated by thyroid hormones [64]. Epb41l5 is implicated in the posttranscriptional regulation of cadherin Cdh1 and cell adhesion molecule Itgb1 during epithelial-mesenchymal transition (EMT) and this transition is inhibited by *Epb41l5* silencing [65].

### Non-intersecting 358 DEG set derived from the comparison group HT *vs*. TN

The predominant overexpression of *IGHV* genes, especially *IGHV3*, that are used for generating autoantibody against TPO is consistent with a review reporting that expression of these genes is associated with HT [66]. Of notice, all *IGHV3* genes in our data compilation were included in the 358 DEG set. Similarly, the CD3 components, which were contained in our data compilation, i.e., *CD3G*, *CD3E*, and *CD3D*, were highly overexpressed in the 358 DEG set. In T cells, these molecules generate activation signals when associated in a complex with the TCR. Therapies with CD3 autoantibodies in combination with other therapeutics are envisaged to improve safety and optimize efficacy for treatment of various autoimmune diseases [67]. PDCD1LG2 is expressed from APCs whereas CD28 is expressed from T cells and both immune checkpoint molecules are implicated in inhibitory and stimulatory T cell signaling, respectively [68]. Also of notice, a pronounced number of critical immunomodulators, which were described in an immunogenomic analysis of major TCGA cancer data sets and included *PDCD1LG2*, *BTN3A2*, *GZMA*, *BTLA*, *CD28*, *ICOS*, and *IDO1*, were contained in the 358 DEG set [69]. The intracellular phosphatase PTPN22 is implicated in immune cell signaling and known as a predisposing gene for autoimmune diseases [70]. A non-synonymous polymorphism in this gene is associated with enhanced susceptibility to a variety of autoimmune diseases [71]. PTPN22 may constitute a target for cancer immunotherapy [70].

### The 950 DEG set from the intersection of all four comparison groups

CXCL10 is known as one of the key immunomodulators in cancer and its high expression upon combined INFG and TNFA stimulation has been demonstrated in primary thyrocyte cell cultures [72]. PARP3 is a member of the poly(ADP-ribose) polymerases and is implicated in biological relevant functions including DNA-repair mechanisms and cell cycle regulation [73]. In *in vitro* experiments, PARP3 expression assisted in TGFB-induced and ROS-supported EMT [74]. PARP3 may gain relevance as a therapeutic target in cancer treatment [75]. An IHC study found a significant association of TP53 positivity in TC with thyroiditis [76]. Increased apoptosis was detected in a case series of HT and thyroid tumors, and it was noted that elevated TP53 and/or CDKN1A protein expression is an indication for possible DNA damage and enhanced apoptosis in HT [77]. Both SOD1 and SOD2, which catalyze the dismutation of superoxide radicals into less reactive hydrogen peroxide and oxygen, were significantly higher expressed in the thyroid lesions compared to TN. A microarray expression analysis of the Oncomine database demonstrated that SOD2 is higher expressed in PTCs and pronounced higher expressed in anaplastic thyroid carcinoma in comparison to normal thyroid tissue [78]. RAD21 is a component of the cohesion core complex that is a critical regulator of sister chromatid cohesion and chromosome segregation. Loss of RAD21 impairs genomic loop domains [79]. Deregulation of RAD21 has been observed in a number of cancers [80]. Different mechanisms of BRCA1 downregulation have been described [81]. Knockdown of *BRAC1* in breast cancer cells resulted in increased phospho-Akt and enhanced susceptibility of the cells to PI3K/AKT pathway inhibitors [82]. The secreted prolymphangiogenic factor VEGFD is implicated in tumor growth; however, decreased serum levels of VEGFD were measured in patients with metastatic differentiated thyroid carcinoma [83].

## Conclusion

Taken together, this microarray expression study provides a comprehensive overview on DEGs and biofunctions in the thyroid lesions under investigation with a primary focus on those that are shared between HT and PTC w/ HT. Our findings are in line with the conception that in HT a lymphocytic infiltrate is associated with chronic exposure to ROS that promotes DNA damage resulting in oncogenic transformation of thyroid cells. The comprehensive lists of DEGs and biofunctions may serve as a source to assess therapeutic targets and biomarkers, especially in HT and PTC w/ HT.

## Supporting information

S1 Fig Hierarchical cluster analysis on the expression values of the 71 DEG set from the intersection of HT *vs*. TN ∩ PTC w/ HT *vs*. TN. Expression values were plotted for all 36 samples analyzed in this study, i.e., 13 HT, eight PTCs w/ HT, six PTCs w/o HT, six mPTCs, and three TN samples. Red and green colors display higher and lower expression values of samples, respectively.
(TIF)

S1 Table Clinicopathological and demographic data of the case series. (XLSX)

S2 Table P-values and fold change of the 15 intersecting and non-intersecting DEG sets derived from the four comparison groups HT vs. TN; PTC w/ HT vs. TN; PTC w/o HT vs. TN; and mPTC vs. TN. (XLSX)

S3 Table Genes listed in different gene ontologies. (XLSX)

## References

[1] Caturegli P, De Remigis A, Rose NR. Hashimoto thyroiditis: clinical and diagnostic criteria. Autoimmunity reviews. 2014;13(4–5):391–7. Epub 01/13. 10.1016/j.autrev.2014.01.007 . 24434360

[2] Rydzewska M, Jaromin M, Pasierowska IE, Stozek K, Bossowski A. Role of the T and B lymphocytes in pathogenesis of autoimmune thyroid diseases. Thyroid research. 2018;11:2 Epub 2018/02/17. 10.1186/s13044-018-0046-9 29449887 PMC5812228

[3] Zaletel K, Gaberšček S. Hashimoto's Thyroiditis: From Genes to the Disease. Current genomics. 2011;12(8):576–88. Epub 2012/06/02. 10.2174/138920211798120763 22654557 PMC3271310

[4] Dong YH, Fu DG. Autoimmune thyroid disease: mechanism, genetics and current knowledge. European review for medical and pharmacological sciences. 2014;18(23):3611–8. Epub 2014/12/24. . 25535130

[5] Balazs C. [The role of hereditary and environmental factors in autoimmune thyroid diseases]. Orvosi hetilap. 2012;153(26):1013–22. Epub 2012/06/28. 10.1556/OH.2012.29370 . 22735372

[6] Hwangbo Y, Park YJ. Genome-Wide Association Studies of Autoimmune Thyroid Diseases, Thyroid Function, and Thyroid Cancer. Endocrinology and metabolism (Seoul, Korea). 2018;33(2):175–84. Epub 2018/06/28. 10.3803/EnM.2018.33.2.175 29947174 PMC6021314

[7] Brcic L, Baric A, Gracan S, Torlak V, Brekalo M, Skrabic V, et al Genome-wide association analysis suggests novel loci underlying thyroid antibodies in Hashimoto's thyroiditis. Scientific reports. 2019;9(1):5360 Epub 2019/03/31. 10.1038/s41598-019-41850-6 30926877 PMC6440971

[8] McGrogan A, Seaman HE, Wright JW, de Vries CS. The incidence of autoimmune thyroid disease: a systematic review of the literature. Clinical endocrinology. 2008;69(5):687–96. 10.1111/j.1365-2265.2008.03338.x . 18673466

[9] Lai X, Xia Y, Zhang B, Li J, Jiang Y. A meta-analysis of Hashimoto's thyroiditis and papillary thyroid carcinoma risk. Oncotarget. 2017;8(37):62414–24. Epub 2017/10/06. 10.18632/oncotarget.18620 28977955 PMC5617515

[10] Ferrari SM, Fallahi P, Elia G, Ragusa F, Ruffilli I, Paparo SR, et al Thyroid autoimmune disorders and cancer. Seminars in cancer biology. 2019 Epub 2019/06/04. 10.1016/j.semcancer.2019.05.019 . 31158464

[11] La Vecchia C, Malvezzi M, Bosetti C, Garavello W, Bertuccio P, Levi F, et al Thyroid cancer mortality and incidence: a global overview. International journal of cancer. 2015;136(9):2187–95. Epub 2014/10/07. 10.1002/ijc.29251 . 25284703

[12] Fatourechi V. Managing the increasing diagnosis of papillary micro-cancer of thyroid. Expert review of endocrinology & metabolism. 2015;10(5):467–9. Epub 2015/09/01. 10.1586/17446651.2015.1063996 . 30298758

[13] Hahn LD, Kunder CA, Chen MM, Orloff LA, Desser TS. Indolent thyroid cancer: knowns and unknowns. Cancers of the head & neck. 2017;2:1 Epub 2017/01/11. 10.1186/s41199-016-0021-x 31093348 PMC6460732

[14] Ito Y, Miyauchi A, Oda H. Low-risk papillary microcarcinoma of the thyroid: A review of active surveillance trials. European journal of surgical oncology: the journal of the European Society of Surgical Oncology and the British Association of Surgical Oncology. 2018;44(3):307–15. Epub 2017/03/28. 10.1016/j.ejso.2017.03.004 . 28343733

[15] Resende de Paiva C, Gronhoj C, Feldt-Rasmussen U, von Buchwald C. Association between Hashimoto's Thyroiditis and Thyroid Cancer in 64,628 Patients. Frontiers in Oncology. 2017;7:53 Epub 2017/04/27. 10.3389/fonc.2017.00053 28443243 PMC5385456

[16] Moon S, Chung HS, Yu JM, Yoo HJ, Park JH, Kim DS, et al Associations between Hashimoto Thyroiditis and Clinical Outcomes of Papillary Thyroid Cancer: A Meta-Analysis of Observational Studies. Endocrinology and metabolism (Seoul, Korea). 2018;33(4):473–84. Epub 2018/12/05. 10.3803/EnM.2018.33.4.473 30513562 PMC6279904

[17] Yi JW, Park JY, Sung JY, Kwak SH, Yu J, Chang JH, et al Genomic evidence of reactive oxygen species elevation in papillary thyroid carcinoma with Hashimoto thyroiditis. Endocrine journal. 2015;62(10):857–77. Epub 2015/07/28. 10.1507/endocrj.EJ15-0234 . 26211532

[18] Ma H, Yan J, Zhang C, Qin S, Qin L, Liu L, et al Expression of papillary thyroid carcinoma-associated molecular markers and their significance in follicular epithelial dysplasia with papillary thyroid carcinoma-like nuclear alterations in Hashimoto's thyroiditis. International journal of clinical and experimental pathology. 2014;7(11):7999–8007. Epub 2015/01/01. 25550843 PMC4270599

[19] Royer MC, Zhang H, Fan CY, Kokoska MS. Genetic alterations in papillary thyroid carcinoma and hashimoto thyroiditis: An analysis of hOGG1 loss of heterozygosity. Archives of otolaryngology—head & neck surgery. 2010;136(3):240–2. 10.1001/archoto.2010.20 . 20231640

[20] Larson SD, Jackson LN, Riall TS, Uchida T, Thomas RP, Qiu S, et al Increased incidence of well-differentiated thyroid cancer associated with Hashimoto thyroiditis and the role of the PI3k/Akt pathway. Journal of the American College of Surgeons. 2007;204(5):764–75. Epub 2007/02/23. 10.1016/j.jamcollsurg.2006.12.037 . 17481480 PMC2430882

[21] Unger P, Ewart M, Wang BY, Gan L, Kohtz DS, Burstein DE. Expression of p63 in papillary thyroid carcinoma and in Hashimoto's thyroiditis: a pathobiologic link? Human pathology. 2003;34(8):764–9. Epub 2003/09/25. 10.1016/s0046-8177(03)00239-9 . 14506636

[22] Rhoden KJ, Unger K, Salvatore G, Yilmaz Y, Vovk V, Chiappetta G, et al RET/papillary thyroid cancer rearrangement in nonneoplastic thyrocytes: follicular cells of Hashimoto's thyroiditis share low-level recombination events with a subset of papillary carcinoma. The Journal of clinical endocrinology and metabolism. 2006;91(6):2414–23. Epub 2006/04/06. 10.1210/jc.2006-0240 . 16595592

[23] Kim KH, Suh KS, Kang DW, Kang DY. Mutations of the BRAF gene in papillary thyroid carcinoma and in Hashimoto's thyroiditis. Pathology international. 2005;55(9):540–5. Epub 2005/09/07. 10.1111/j.1440-1827.2005.01866.x . 16143028

[24] Schulten HJ, Alotibi R, Al-Ahmadi A, Ata M, Karim S, Huwait E, et al Effect of BRAF mutational status on expression profiles in conventional papillary thyroid carcinomas. BMC genomics. 2015;16 Suppl 1:S6 Epub 2015/04/30. 10.1186/1471-2164-16-s1-s6 25922907 PMC4315163

[25] Schulten HJ, Salama S, Al-Mansouri Z, Alotibi R, Al-Ghamdi K, Al-Hamour OA, et al BRAF mutations in thyroid tumors from an ethnically diverse group. Hereditary cancer in clinical practice. 2012;10(1):10 Epub 2012/08/29. 10.1186/1897-4287-10-10 22925390 PMC3434056

[26] Hunt SE, McLaren W, Gil L, Thormann A, Schuilenburg H, Sheppard D, et al Ensembl variation resources. Database: the journal of biological databases and curation. 2018;2018 Epub 2018/12/24. 10.1093/database/bay119 30576484 PMC6310513

[27] Braschi B, Denny P, Gray K, Jones T, Seal R, Tweedie S, et al Genenames.org: the HGNC and VGNC resources in 2019. Nucleic acids research. 2019;47(D1):D786–d92. Epub 2018/10/12. 10.1093/nar/gky930 30304474 PMC6324057

[28] Babicki S, Arndt D, Marcu A, Liang Y, Grant JR, Maciejewski A, et al Heatmapper: web-enabled heat mapping for all. Nucleic acids research. 2016;44(W1):W147–53. Epub 2016/05/18. 10.1093/nar/gkw419 27190236 PMC4987948

[29] The Gene Ontology Resource: 20 years and still GOing strong. Nucleic acids research. 2019;47(D1):D330–d8. Epub 2018/11/06. 10.1093/nar/gky1055 30395331 PMC6323945

[30] Liberzon A, Birger C, Thorvaldsdottir H, Ghandi M, Mesirov JP, Tamayo P. The Molecular Signatures Database (MSigDB) hallmark gene set collection. Cell systems. 2015;1(6):417–25. Epub 2016/01/16. 10.1016/j.cels.2015.12.004 26771021 PMC4707969

[31] Chae YK, Anker JF, Carneiro BA, Chandra S, Kaplan J, Kalyan A, et al Genomic landscape of DNA repair genes in cancer. Oncotarget. 2016;7(17):23312–21. Epub 2016/03/24. 10.18632/oncotarget.8196 27004405 PMC5029628

[32] Andres-Leon E, Cases I, Arcas A, Rojas AM. DDRprot: a database of DNA damage response-related proteins. Database: the journal of biological databases and curation. 2016;2016 Epub 2016/09/01. 10.1093/database/baw123 27577567 PMC5004197

[33] Gao J, Aksoy BA, Dogrusoz U, Dresdner G, Gross B, Sumer SO, et al Integrative analysis of complex cancer genomics and clinical profiles using the cBioPortal. Science signaling. 2013;6(269):pl1 Epub 2013/04/04. 10.1126/scisignal.2004088 23550210 PMC4160307

[34] Tanaka T, Umeki K, Yamamoto I, Sugiyama S, Noguchi S, Ohtaki S. Immunohistochemical loss of thyroid peroxidase in papillary thyroid carcinoma: strong suppression of peroxidase gene expression. The Journal of pathology. 1996;179(1):89–94. Epub 1996/05/01. 10.1002/(SICI)1096-9896(199605)179:1<89::AID-PATH546>3.0.CO;2-R . 8691351

[35] Reuter S, Gupta SC, Chaturvedi MM, Aggarwal BB. Oxidative stress, inflammation, and cancer: how are they linked? Free radical biology & medicine. 2010;49(11):1603–16. Epub 2010/09/16. 10.1016/j.freeradbiomed.2010.09.006 20840865 PMC2990475

[36] Qiu Y, Korteweg C, Chen Z, Li J, Luo J, Huang G, et al Immunoglobulin G expression and its colocalization with complement proteins in papillary thyroid cancer. Modern pathology: an official journal of the United States and Canadian Academy of Pathology, Inc. 2012;25(1):36–45. 10.1038/modpathol.2011.139 . 21909078

[37] Ehlers M, Schott M. Hashimoto's thyroiditis and papillary thyroid cancer: are they immunologically linked? Trends in endocrinology and metabolism: TEM. 2014;25(12):656–64. Epub 2014/10/14. 10.1016/j.tem.2014.09.001 . 25306886

[38] Zeppa P, Cozzolino I, Peluso AL, Troncone G, Lucariello A, Picardi M, et al Cytologic, flow cytometry, and molecular assessment of lymphoid infiltrate in fine-needle cytology samples of Hashimoto thyroiditis. Cancer. 2009;117(3):174–84. 10.1002/cncy.20022 . 19382168

[39] Groot Kormelink T, Powe DG, Kuijpers SA, Abudukelimu A, Fens MH, Pieters EH, et al Immunoglobulin free light chains are biomarkers of poor prognosis in basal-like breast cancer and are potential targets in tumor-associated inflammation. Oncotarget. 2014;5(10):3159–67. Epub 2014/06/17. 10.18632/oncotarget.1868 24931643 PMC4102799

[40] Finn SP, Smyth P, Cahill S, Streck C, O'Regan EM, Flavin R, et al Expression microarray analysis of papillary thyroid carcinoma and benign thyroid tissue: emphasis on the follicular variant and potential markers of malignancy. Virchows Archiv: an international journal of pathology. 2007;450(3):249–60. 10.1007/s00428-006-0348-5 17252232 PMC1888716

[41] Smith MJ, Rihanek M, Coleman BM, Gottlieb PA, Sarapura VD, Cambier JC. Activation of thyroid antigen-reactive B cells in recent onset autoimmune thyroid disease patients. Journal of autoimmunity. 2018;89:82–9. Epub 2017/12/14. 10.1016/j.jaut.2017.12.001 29233566 PMC5902436

[42] Yeh SJ, Lin CY, Li CW, Chen BS. Systems Biology Approaches to Investigate Genetic and Epigenetic Molecular Progression Mechanisms for Identifying Gene Expression Signatures in Papillary Thyroid Cancer. International journal of molecular sciences. 2019;20(10). Epub 2019/05/28. 10.3390/ijms20102536 31126066 PMC6566633

[43] Schoggins JW. Recent advances in antiviral interferon-stimulated gene biology. F1000Research. 2018;7:309 Epub 2018/03/24. 10.12688/f1000research.12450.1 29568506 PMC5850085

[44] Morgan AA, Pyrgos VJ, Nadeau KC, Williamson PR, Butte AJ. Multiplex meta-analysis of RNA expression to identify genes with variants associated with immune dysfunction. Journal of the American Medical Informatics Association: JAMIA. 2012;19(2):284–8. Epub 2012/02/10. 10.1136/amiajnl-2011-000657 22319178 PMC3277634

[45] Kimura HJ, Rocchi R, Landek-Salgado MA, Suzuki K, Chen CY, Kimura M, et al Influence of signal transducer and activator of transcription-1 signaling on thyroid morphology and function. Endocrinology. 2009;150(7):3409–16. Epub 2009/03/28. 10.1210/en.2008-1769 19325004 PMC2703527

[46] Staab J, Barth PJ, Meyer T. Cell-type-specific expression of STAT transcription factors in tissue samples from patients with lymphocytic thyroiditis. Endocrine pathology. 2012;23(3):141–50. Epub 2012/04/25. 10.1007/s12022-012-9204-0 22527947 PMC3417099

[47] de Prati AC, Ciampa AR, Cavalieri E, Zaffini R, Darra E, Menegazzi M, et al STAT1 as a new molecular target of anti-inflammatory treatment. Current medicinal chemistry. 2005;12(16):1819–28. Epub 2005/08/17. 10.2174/0929867054546645 . 16101503

[48] Hrdinka M, Horejsi V. PAG—a multipurpose transmembrane adaptor protein. Oncogene. 2014;33(41):4881–92. Epub 2013/11/12. 10.1038/onc.2013.485 . 24213579

[49] Jin M. Unique roles of tryptophanyl-tRNA synthetase in immune control and its therapeutic implications. Experimental & molecular medicine. 2019;51(1):1 Epub 2019/01/08. 10.1038/s12276-018-0196-9 30613102 PMC6321835

[50] Du X, Tang Y, Xu H, Lit L, Walker W, Ashwood P, et al Genomic profiles for human peripheral blood T cells, B cells, natural killer cells, monocytes, and polymorphonuclear cells: comparisons to ischemic stroke, migraine, and Tourette syndrome. Genomics. 2006;87(6):693–703. Epub 2006/03/21. 10.1016/j.ygeno.2006.02.003 . 16546348

[51] Lim MB, Kuiper JW, Katchky A, Goldberg H, Glogauer M. Rac2 is required for the formation of neutrophil extracellular traps. Journal of leukocyte biology. 2011;90(4):771–6. Epub 2011/06/30. 10.1189/jlb.1010549 . 21712395

[52] Joshi S, Singh AR, Zulcic M, Bao L, Messer K, Ideker T, et al Rac2 controls tumor growth, metastasis and M1-M2 macrophage differentiation in vivo. PloS one. 2014;9(4):e95893 Epub 2014/04/29. 10.1371/journal.pone.0095893 24770346 PMC4000195

[53] Thomas DC. The phagocyte respiratory burst: Historical perspectives and recent advances. Immunology letters. 2017;192:88–96. Epub 2017/09/03. 10.1016/j.imlet.2017.08.016 . 28864335

[54] Kalhori V, Tornquist K. MMP2 and MMP9 participate in S1P-induced invasion of follicular ML-1 thyroid cancer cells. Mol Cell Endocrinol. 2015;404:113–22. Epub 2015/02/04. 10.1016/j.mce.2015.01.037 . 25643979

[55] Luo D, Chen H, Li X, Lu P, Long M, Peng X, et al Activation of the ROCK1/MMP-9 pathway is associated with the invasion and poor prognosis in papillary thyroid carcinoma. International journal of oncology. 2017;51(4):1209–18. Epub 2017/08/30. 10.3892/ijo.2017.4100 . 28848996

[56] Abramson J, Giraud M, Benoist C, Mathis D. Aire's partners in the molecular control of immunological tolerance. Cell. 2010;140(1):123–35. Epub 2010/01/21. 10.1016/j.cell.2009.12.030 . 20085707

[57] Wong YL, Anzola JV, Davis RL, Yoon M, Motamedi A, Kroll A, et al Cell biology. Reversible centriole depletion with an inhibitor of Polo-like kinase 4. Science (New York, NY). 2015;348(6239):1155–60. Epub 2015/05/02. 10.1126/science.aaa5111 25931445 PMC4764081

[58] Liao GB, Li XZ, Zeng S, Liu C, Yang SM, Yang L, et al Regulation of the master regulator FOXM1 in cancer. Cell communication and signaling: CCS. 2018;16(1):57 Epub 2018/09/14. 10.1186/s12964-018-0266-6 30208972 PMC6134757

[59] Engeland K. Cell cycle arrest through indirect transcriptional repression by p53: I have a DREAM. Cell death and differentiation. 2018;25(1):114–32. Epub 2017/11/11. 10.1038/cdd.2017.172 29125603 PMC5729532

[60] Ahmed M, Uddin S, Hussain AR, Alyan A, Jehan Z, Al-Dayel F, et al FoxM1 and its association with matrix metalloproteinases (MMP) signaling pathway in papillary thyroid carcinoma. The Journal of Clinical Endocrinology and Metabolism. 2012;97(1):E1–E13. 10.1210/jc.2011-1506 . 22049175

[61] Wilson IM, Vucic EA, Enfield KS, Thu KL, Zhang YA, Chari R, et al EYA4 is inactivated biallelically at a high frequency in sporadic lung cancer and is associated with familial lung cancer risk. Oncogene. 2014;33(36):4464–73. Epub 2013/10/08. 10.1038/onc.2013.396 24096489 PMC4527534

[62] Aust G, Zhu D, Van Meir EG, Xu L. Adhesion GPCRs in Tumorigenesis. Handbook of experimental pharmacology. 2016;234:369–96. Epub 2016/11/11. 10.1007/978-3-319-41523-9_17 27832497 PMC5389670

[63] Hassan A, Bagu ET, Levesque M, Patten SA, Benhadjeba S, Edjekouane L, et al The 17beta-estradiol induced upregulation of the adhesion G-protein coupled receptor (ADGRG7) is modulated by ESRalpha and SP1 complex. Biology open. 2019;8(1). Epub 2019/01/02. 10.1242/bio.037390 30598481 PMC6361214

[64] Sinha RA, Singh BK, Yen PM. Direct effects of thyroid hormones on hepatic lipid metabolism. Nature reviews Endocrinology. 2018;14(5):259–69. Epub 2018/02/24. 10.1038/nrendo.2018.10 29472712 PMC6013028

[65] Hirano M, Hashimoto S, Yonemura S, Sabe H, Aizawa S. EPB41L5 functions to post-transcriptionally regulate cadherin and integrin during epithelial-mesenchymal transition. The Journal of cell biology. 2008;182(6):1217–30. Epub 2008/09/17. 10.1083/jcb.200712086 18794329 PMC2542480

[66] Chardes T, Chapal N, Bresson D, Bes C, Giudicelli V, Lefranc MP, et al The human anti-thyroid peroxidase autoantibody repertoire in Graves' and Hashimoto's autoimmune thyroid diseases. Immunogenetics. 2002;54(3):141–57. Epub 2002/06/20. 10.1007/s00251-002-0453-9 . 12073143

[67] Dean Y, Depis F, Kosco-Vilbois M. Combination therapies in the context of anti-CD3 antibodies for the treatment of autoimmune diseases. Swiss medical weekly. 2012;142:w13711 Epub 2012/12/21. 10.4414/smw.2012.13711 . 23254986

[68] Ulisse S, Tuccilli C, Sorrenti S, Antonelli A, Fallahi P, D'Armiento E, et al PD-1 Ligand Expression in Epithelial Thyroid Cancers: Potential Clinical Implications. International journal of molecular sciences. 2019;20(6). Epub 2019/03/23. 10.3390/ijms20061405 30897754 PMC6471477

[69] Thorsson V, Gibbs DL, Brown SD, Wolf D, Bortone DS, Ou Yang TH, et al The Immune Landscape of Cancer. Immunity. 2018;48(4):812–30.e14. Epub 2018/04/10. 10.1016/j.immuni.2018.03.023 29628290 PMC5982584

[70] Brownlie RJ, Garcia C, Ravasz M, Zehn D, Salmond RJ, Zamoyska R. Resistance to TGFbeta suppression and improved anti-tumor responses in CD8(+) T cells lacking PTPN22. Nature communications. 2017;8(1):1343 Epub 2017/11/09. 10.1038/s41467-017-01427-1 29116089 PMC5676842

[71] Clarke F, Purvis HA, Sanchez-Blanco C, Gutierrez-Martinez E, Cornish GH, Zamoyska R, et al The protein tyrosine phosphatase PTPN22 negatively regulates presentation of immune complex derived antigens. Scientific reports. 2018;8(1):12692 Epub 2018/08/25. 10.1038/s41598-018-31179-x 30139951 PMC6107551

[72] Rotondi M, Coperchini F, Pignatti P, Sideri R, Groppelli G, Leporati P, et al Interferon-gamma and tumor necrosis factor-alpha sustain secretion of specific CXC chemokines in human thyrocytes: a first step toward a differentiation between autoimmune and tumor-related inflammation? The Journal of clinical endocrinology and metabolism. 2013;98(1):308–13. Epub 2012/11/03. 10.1210/jc.2012-2555 . 23118425

[73] Bai P. Biology of Poly(ADP-Ribose) Polymerases: The Factotums of Cell Maintenance. Molecular cell. 2015;58(6):947–58. Epub 2015/06/20. 10.1016/j.molcel.2015.01.034 . 26091343

[74] Karicheva O, Rodriguez-Vargas JM, Wadier N, Martin-Hernandez K, Vauchelles R, Magroun N, et al PARP3 controls TGFbeta and ROS driven epithelial-to-mesenchymal transition and stemness by stimulating a TG2-Snail-E-cadherin axis. Oncotarget. 2016;7(39):64109–23. Epub 2016/09/01. 10.18632/oncotarget.11627 27579892 PMC5325429

[75] Rodriguez-Vargas JM, Nguekeu-Zebaze L, Dantzer F. PARP3 comes to light as a prime target in cancer therapy. Cell cycle (Georgetown, Tex). 2019;18(12):1295–301. Epub 2019/05/17. 10.1080/15384101.2019.1617454 31095444 PMC6592235

[76] Marcello MA, Morari EC, Cunha LL, De Nadai Silva AC, Carraro DM, Carvalho AL, et al P53 and expression of immunological markers may identify early stage thyroid tumors. Clinical & developmental immunology. 2013;2013:846584 Epub 2013/10/31. 10.1155/2013/846584 24171036 PMC3792533

[77] Okayasu I, Osakabe T, Onozawa M, Mikami T, Fujiwara M. p53 and p21(WAF1) expression in lymphocytic thyroiditis and thyroid tumors. Clinical immunology and immunopathology. 1998;88(2):183–91. Epub 1998/08/26. 10.1006/clin.1998.4572 . 9714696

[78] Cammarota F, Fiscardi F, Esposito T, de Vita G, Salvatore M, Laukkanen MO. Clinical relevance of thyroid cell models in redox research. Cancer cell international. 2015;15:113 Epub 2015/12/15. 10.1186/s12935-015-0264-3 26664298 PMC4673788

[79] Rao SSP, Huang SC, Glenn St Hilaire B, Engreitz JM, Perez EM, Kieffer-Kwon KR, et al Cohesin Loss Eliminates All Loop Domains. Cell. 2017;171(2):305–20.e24. Epub 2017/10/07. 10.1016/j.cell.2017.09.026 28985562 PMC5846482

[80] O'Neil NJ, van Pel DM, Hieter P. Synthetic lethality and cancer: cohesin and PARP at the replication fork. Trends in genetics: TIG. 2013;29(5):290–7. Epub 2013/01/22. 10.1016/j.tig.2012.12.004 23333522 PMC3868440

[81] Gorodetska I, Kozeretska I, Dubrovska A. BRCA Genes: The Role in Genome Stability, Cancer Stemness and Therapy Resistance. Journal of Cancer. 2019;10(9):2109–27. Epub 2019/06/18. 10.7150/jca.30410 31205572 PMC6548160

[82] Yi YW, Kang HJ, Kim HJ, Hwang JS, Wang A, Bae I. Inhibition of constitutively activated phosphoinositide 3-kinase/AKT pathway enhances antitumor activity of chemotherapeutic agents in breast cancer susceptibility gene 1-defective breast cancer cells. Molecular carcinogenesis. 2013;52(9):667–75. Epub 2012/04/11. 10.1002/mc.21905 22488590 PMC3586771

[83] Nersita R, Matrone A, Klain M, Scavuzzo F, Vitolo G, Abbondanza C, et al Decreased serum vascular endothelial growth factor-D levels in metastatic patients with differentiated thyroid carcinoma. Clinical endocrinology. 2012;76(1):142–6. Epub 2011/07/26. 10.1111/j.1365-2265.2011.04183.x . 21781145

