# Supplementary figures and images for: Genetic relationship between Hashimoto`s thyroiditis and papillary thyroid carcinoma with coexisting Hashimoto`s thyroiditis
Source: PLoS One. 2020 Jun 30;15(6):e0234566. doi: 10.1371/journal.pone.0234566 (PMC7326236; doi:10.1371/journal.pone.0234566)

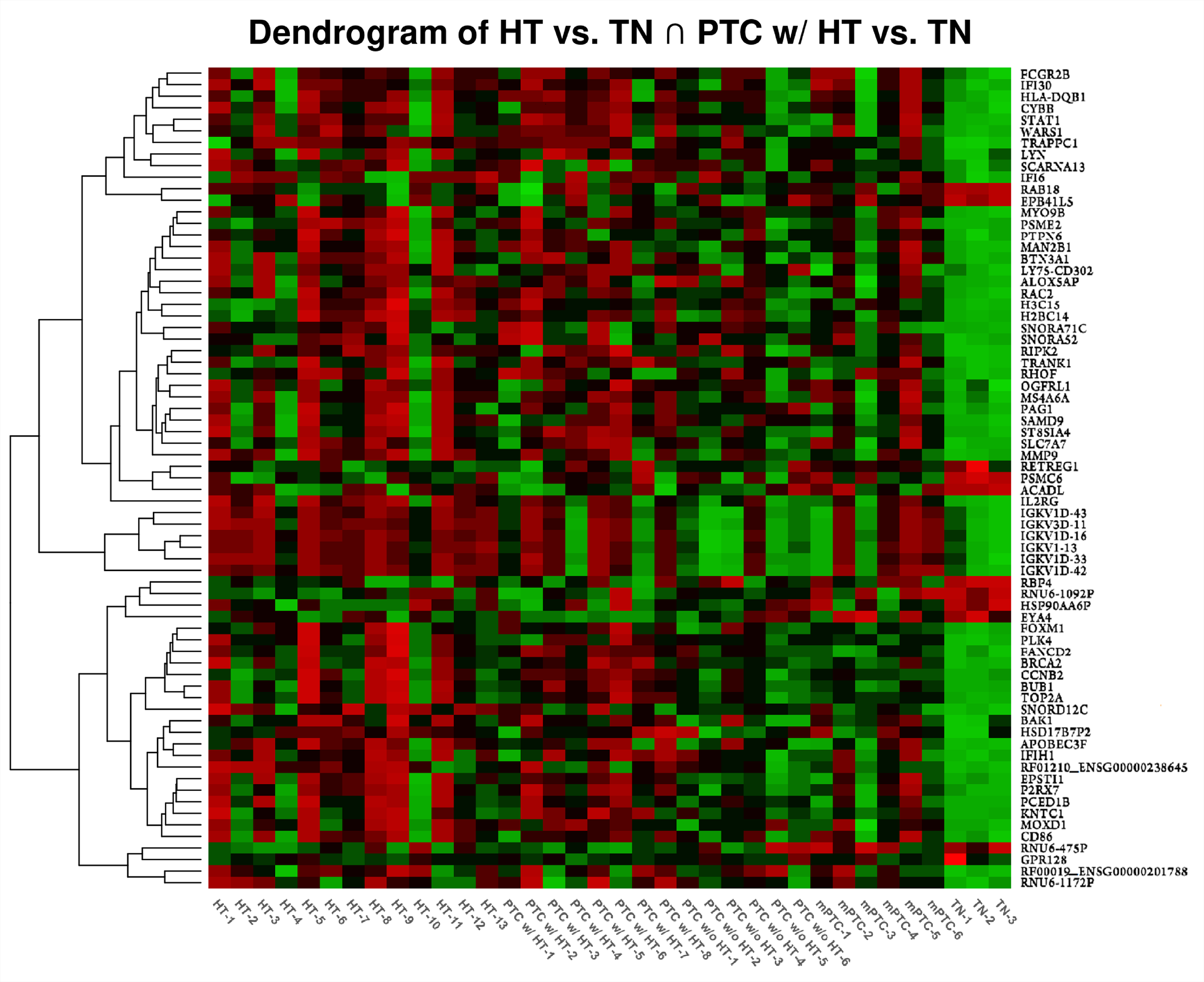

Supplement: S1 Fig — Expression values were plotted for all 36 samples analyzed in this study, i.e., 13 HT, eight PTCs w/ HT, six PTCs w/o HT, six mPTCs, and three TN samples. Red and green colors display higher and lower expression values of samples, respectively. (TIF) [file pone.0234566.s001.tif]
